# Eleven-month SARS-CoV-2 binding antibody decay, and associated factors, among mRNA vaccinees: implications for booster vaccination

**DOI:** 10.1099/acmi.0.000678.v3

**Published:** 2023-11-28

**Authors:** Michael Asamoah-Boaheng, Brian Grunau, Scott Haig, Mohammad Ehsanul Karim, Tracy Kirkham, Pascal M. Lavoie, Sadaf Sediqi, Steven J. Drews, Sheila F. O'Brien, Vilte Barakauskas, Ana Citlali Marquez, Agatha Jassem, David M. Goldfarb

**Affiliations:** ^1^​ Department of Emergency Medicine, University of British Columbia, Vancouver, British Columbia, Canada; ^2^​ Centre for Advancing Health Outcomes, University of British Columbia, Vancouver, British Columbia, Canada; ^3^​ School of Population and Public Health, University of British Columbia, Vancouver, British Columbia, Canada; ^4^​ British Columbia Emergency Health Services, Vancouver, British Columbia, Canada; ^5^​ Dalla Lana School of Public Health, University of Toronto, Toronto, Ontario, Canada; ^6^​ Department of Pediatrics, University of British Columbia, Vancouver, British Columbia, Canada; ^7^​ Department of Pathology and Laboratory Medicine, University of British Columbia, Vancouver, British Columbia, Canada; ^8^​ Canadian Blood Services, Vancouver, British Columbia, Canada; ^9^​ Division of Diagnostic and Applied Microbiology, Laboratory Medicine and Pathology, University of Alberta, Alberta, Canada; ^10^​ School of Epidemiology & Public Health, University of Ottawa, Ottawa, Ontario, Canada; ^11^​ Public Health Laboratory, British Columbia Centre for Disease Control, Vancouver, British Columbia, Canada; ^12^​ British Columbia Children’s Hospital Research Institute, British Columbia Children’s Hospital, Vancouver, British Columbia, Canada

**Keywords:** SARS-CoV-2, immunogenicity decay, risk factors, antibody levels, mRNA COVID-19 vaccines

## Abstract

**Background.:**

We examined the 11 month longitudinal antibody decay among two-dose mRNA vaccinees, and identified factors associated with faster decay.

**Methods.:**

The study included samples from the COVID-19 Occupational Risk, Seroprevalence and Immunity among Paramedics (CORSIP) longitudinal observational study of paramedics in Canada. Participants were included if they had received two mRNA vaccines without prior SARS-CoV-2 infection and provided two blood samples post-vaccination. The outcomes of interest were quantitative SARS-CoV-2 antibody concentrations. We employed spaghetti and scatter plots (with kernel-weighted local polynomial smoothing curve) to describe the trend of the antibody decay over 11 months post-vaccine and fit a mixed effect exponential decay model to examine the loss of immunogenicity and factors associated with antibody waning over time.

**Results.:**

This analysis included 652 blood samples from 326 adult paramedics. Total anti-spike antibody levels peaked on the twenty-first day (antibody level 9042 U ml^−1^) after the second mRNA vaccine dose. Total anti-spike antibody levels declined thereafter, with a half-life of 94 [95 % CI: 70, 143] days, with levels plateauing at 295 days (antibody level 1021 U ml^−1^). Older age, vaccine dosing interval <35 days, and the BNT162b2 vaccine (compared to mRNA-1273 vaccine) were associated with faster antibody decay.

**Conclusion.:**

Antibody levels declined after the initial mRNA series with a half-life of 94 days, plateauing at 295 days. These findings may inform the timing of booster vaccine doses and identifying individuals with faster antibody decay.

## Data Summary statement

The CORSIP data used for this study can be publicly assessed through the website of Canada COVID-19 Immunity Task Force (CITF) website via the link: https://portal.citf.mcgill.ca/.

## Introduction

Data from observational studies and randomized controlled trials have demonstrated the effectiveness of COVID-19 vaccines against symptomatic illness, COVD-19-related hospitalizations, complications, and death [1–4]. However, the long-term duration of vaccine-induced immunity is unclear. Previous studies document waning of SARS-CoV-2 antibody levels after both vaccination and infection [5], with anti-spike levels decreasing substantially as early as 3 months after the second BNT162b2 dose [6]. Humoral responses have been shown to be significantly decreased 6 months after the receipt of the second dose of BNT162b2 vaccine, especially among older adults (≥ 65 years) and those with compromised immune systems [6–11]. However, data on the long-term (>6 month) immunogenicity post-SARS-CoV-2 vaccination remain scarce [6, 7, 10, 11].

Given evidence of waning SARS-CoV-2 humoral immune responses after vaccination, booster vaccinations have been implemented in many jurisdictions; however, the optimal timing for boosters remains unclear. There is growing evidence that SARS-CoV-2 antibody levels provide a measure of COVID-19 risk [12–14] and COVID-19 severity [15–17], a relationship that has been shown to be present even within the Omicron era [18]. Thus, antibody levels may inform decisions regarding the optimal timing of a booster vaccination. Further, given that immunity has been shown to differ based on individual characteristics, it is possible that the optimal booster schedule may vary within different patient groups. Currently there is limited data showing the long-term durability of, and factors associated with, antibody levels post-vaccination. We sought to investigate antibody waning 11 months after two mRNA vaccine doses among adult COVID-19 naïve paramedics in Canada, and factors associated with these outcomes.

## Methods

### Study setting, design, and ethics

Our study included samples of paramedics from the COVID-19 Occupational Risks, Seroprevalence, and Immunity among Paramedics in Canada (CORSIP) study [19]. CORSIP is a longitudinal observational study investigating the seroprevalence of SARS-CoV-2 antibodies among adult (≥ 19 years) Canadian paramedics. Participants provided blood samples and data from structured questionnaires on vaccination and COVID-19 history, past medical history, demographic and workplace characteristics.

### Study participants

For this investigation, we included participants who provided two blood samples (at different times) after receiving only two mRNA vaccines of the same type (either two doses of BNT162b2, or two doses of mRNA-1273 vaccines). We excluded participants who had evidence of prior SARS-CoV-2 infection at any time prior to the second blood collection, based on reported positive nucleic acid amplification viral testing or a reactive blood sample on the Elecsys Nucleocapsid Anti-SARS-CoV-2 (Roche, IND, USA) assay [20]. The CORSIP data used for this study can be publicly assessed through the website of Canada COVID-19 Immunity Task Force (CITF) website via the link: https://portal.citf.mcgill.ca/.

### Serological testing

We tested all samples with: (1) Elecsys Anti-SARS-CoV-2 (nucleocapsid) (Roche Diagnostics International Ltd, Rotkreuz, Switzerland) assay [20, 21] to confirm eligibility; (2) the quantitative Roche Elecsys Anti-SARS-CoV-2 (S) (Roche Diagnostics International Ltd, Rotkreuz, Switzerland) assay for measuring spike total antibody concentrations; and (3) the Meso Scale Discovery (MSD) V-PLEX COVID-19 Coronavirus Panel 2 IgG assay for measuring IgG to spike and receptor-binding domain (RBD) antigens.

### Study outcomes

The primary outcome was total anti-spike antibody concentrations (measured with the Elecsys assay), and the secondary outcomes were IgG concentrations to spike and RBD antigens (measured with the VPLEX assay).

### Statistical analysis

We described continuous variables with mean and standard deviation (SD) for near normally distributed variables without any influential outliers, or median (with interquartile range [IQR]) for skewed or non-normally distributed variables. Categorical variables were described with counts and percentages. Antibody concentrations (including: total anti-spike, anti-spike IgG and anti-RBD IgG antibody concentrations) were presented as geometric mean (GM) with corresponding geometric standard deviations (GSD). We described the longitudinal changes in SARS-COV-2 antibodies 11 months after the second mRNA vaccine dose with scatter (with kernel-weighted local polynomial smoothing curve) [22, 23] and spaghetti plots. Using the kernel-weighted local polynomial smoothing approach with Epanechnikov kernel function [22], we generated the smoothing values and their corresponding smoothing grids and estimated the peak antibody concentration based on the maximum kernel-weighted values. The smoothing grid (days after the second vaccine) that corresponded to the maximum kernel-weighted smoothing value was considered as the day of the peak antibody level. We used the double exponential decay (DED) model [24] to determine the time at which the antibody level stopped declining (the ‘plateau level’) [See supplementary material].

To further demonstrate differences in antibody levels after vaccination, we categorized samples into quartiles based on the number of days they were collected after the second vaccine dose, and plotted box-and-whisker plots to diagram antibody levels.

We modelled the persistence of antibody levels over time using a mixed effect exponential decay (ED) model. The mean structure of the exponential decay model with random intercept and slope is given by:

$${log}_{10}\left({Ab}_{i,j}\right)=\left(\beta_{0}+b_{0i}\right)+\left(\beta_{1}+b_{1i}\right).T_{i,j}+{Ɛ}_{i,j}$$

$\beta_{0}$ and $\beta_{1}$ are the fixed effects intercept and decay rate respectively, while $b_{0i}$ and $b_{1i}$ are the subject-specific (random effects) intercept and decay rates respectively. ${Ɛ}_{i,j}$ represents the random error term for participant ‘*i*´ at time (day) ‘*j*’ which is assumed to be normally distributed; ${log}_{10}\left({Ab}_{i,j}\right)$ is the mean log antibody titre at time $T_{i,j}$ post vaccination [9, 25]. To determine the waning of antibody levels over time, we used the mixed effects ED model to estimate the half-life (the time the peak antibody level was reduced by 50 %) [9]. Thus, the half-life ( $t_{1/2})$ was estimated as by [25]):

$$t_{1/2}=\frac{{log}_{10}(0.5)}{\beta_{1}}$$

Further, we fit a mixed effect ED model to investigate the factors associated with antibody decay over the 11 month study observation period. The mixed effect ED model with random intercept was used to account for the repeated measurements of antibody concentrations for each participant at the two different time points. This model has been used in other studies that investigated antibody waning among vaccinated individuals over time [9, 25]. The various factors included in the model were: participant age (years, continuous variable), female sex at birth (vs. male); ‘racialized’ (including those who self-described their ethnicity or race as South Asian, Chinese, Black, Filipina, Latin American, Arab, Southeast Asian, West Asian, Korean, or Japanese) (vs. whites); ‘BMI: 18.5 to <25 kg m^−2^ (vs. others)’, ‘BMI≥25 kg m^−2^ (vs. others)’; ‘BNT162b2 vaccine (vs. mRNA-1273)’; ‘short vaccine dosing interval’ (binary variable, ‘short’ defined as a vaccine dosing interval less than the median value); and past medical history (including covariates: hypertension, diabetes, asthma, liver diseases, and cancer).

## Results

The study included 652 samples from 326 paramedics, with a mean age of 42 (SD=11) years, where 46 % were female. The majority of the study participants (82 %) were vaccinated with two doses of BNT162b2 vaccine while the remaining 18 % received two doses of mRNA-1273.

Table 1 shows patient characteristics, intervals between vaccines and blood collection dates, and outcome measures. The first and second blood collection occurred at a median of 59 (IQR 29, 94) and 156 (IQR 145, 176) days after the second vaccine dose, respectively. The GM (GSD) of the total anti-spike antibody concentration at the first and second blood collection was 2940 (3.5) U ml^−1^ and 1455 (2.4) U ml^−1^, respectively; for anti-spike IgG was 102 051 (3.1) AU ml^−1^ and 30 956 (2.0) AU ml^−1^, and for anti-spike RBD was 66 986 (3.8) AU ml^−1^ and 17 406 (2.3) AU ml^−1^, respectively.

**Table 1.** Participants characteristics

Variables	N (%) or Mean (SD) or Median (IQR)
***Baseline characteristics***	***N*=326 (*at baseline***)
Age, years, mean (SD)	42 (11)
Female sex (at birth), n (%)	151 (46)
Racialized	25 (7.7)
Body Mass Index (BMI), mean (SD)	27 (5.0)
Obesity (≥ 30 Kg m^−2^), n (%)	88 (27)
Tobacco use, n (%)	13 (4.0)
*Medical History*, n (%)	
Hypertension	30 (9.2)
Diabetes	5.0 (1.5)
Asthma	55 (17)
Chronic Lung Disease	3.0 (0.9)
Heart diseases	1.0 (0.3)
Kidney diseases	1.0 (0.3)
Liver disease	5.0 (1.5)
Cancer	7.0 (2.1)
*Vaccine type*, n (%)	
Pfizer (BNT162b2)	268 (82)
Moderna (mRNA-1273)	60 (18)
*Vaccine doses*, n (%)	
First and second doses (BNT162b2)	266 (82)
First and second doses (mRNA-1273)	60 (18)
Vaccine dosing Interval (days), Median (IQR)	35 (28, 42)
*Time related variables*	
BC 1 date, median (IQR)	16/04/2021 (11/03/2021, 02/06/2021)
BC 2 date, median (IQR)	17/07/2021 (09/07/2021, 25/08/2021)
BC**_1_**-to-BC**_2_** interval (days), Median (IQR)	100 (76, 132)
V**_2_**-to-BC**_1_** interval (days), Median (IQR)	59 (29, 94)
V**_2_**-to-BC**_2_** interval (days), Median (IQR)	156 (145, 176)
***Outcome variables (at follow-up)***	
*Quantitative Antibody Concentrations, GM (GSD)*	
Blood Collection 1	
Anti-Spike total antibody concentration	2940 (3.5)
Anti-Spike IgG concentration	102 051 (3.1)
Anti-RBD IgG concentration	66 986 (3.8)
Blood Collection 2	
Anti-Spike total antibody concentration	1455 (2.4)
Anti-Spike IgG concentration	30 956 (2)
Anti-RBD IgG concentration	17 406 (2.3)

SD: Standard deviation; gMean: geometric mean; gSD: geometric standard deviation; IQR: Interquartile range; BC1, first blood collection date; BC2, second blood collection date; V_2_ : Second vaccine dose date; Vaccine dosing interval, the number of days between V1 and V2; *Racialized*: means other non-white races including Asian ethnic groups, blacks, and others.

**Fig. 1.**
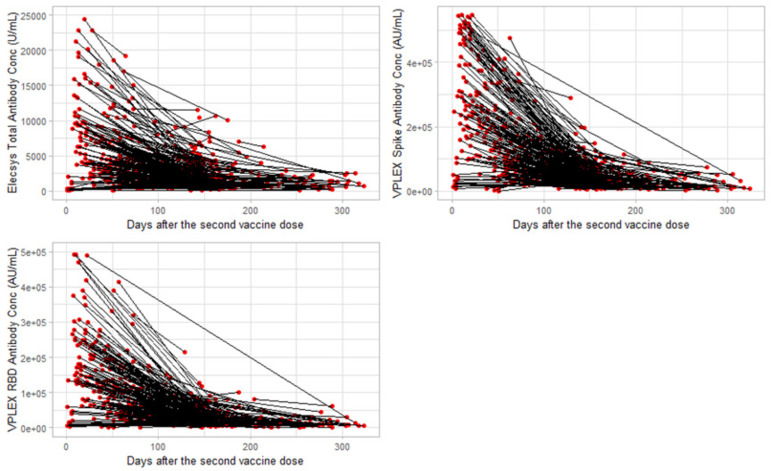
Spaghetti plots of longitudinal changes in antibody concentrations 11 months after two mRNA vaccine doses **Days after second vaccine dose (derived from the timing between second vaccine dose and all blood collections).

**Fig. 2.**
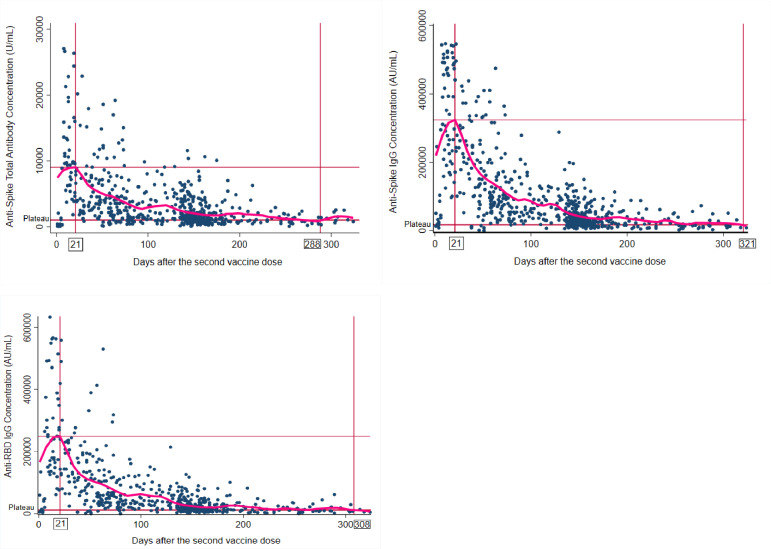
Scatter plots (with kernel-weighted local polynomial smoothing curve) of longitudinal changes in antibody concentrations 11 months after two mRNA vaccine doses. *Vertical lines indicate the time the peak antibody level was recorded, and the time at which the antibody levels plateaued; **horizontal lines indicate the peak antibody level and the value the antibody levels plateaued respectively. **Days after second vaccine dose (derived from the timing between second vaccine dose and all blood collections).

Figs 1, 2 (spaghetti plots) (scatter plots, with smooth curve), and S1 (box plots), describe the longitudinal changes in antibody concentrations during the 11 months after the second vaccine. The peak values for total anti-spike (9 042 U ml^−1^), anti-spike IgG (323 980 AU ml^−1^), and anti-RBD IgG (249 051 AU ml^−1^) antibody concentrations were all recorded on the twenty-first day after the second vaccine dose (Fig. 2 and Table S1, available in the online version of this article). On the two hundred eighty-eighth day after vaccination, total anti-spike antibody levels stopped declining, plateauing at 1021 U ml^−1^ (Fig. 2 and Table S1) which was 11 % of the peak value. Anti-spike IgG and anti-RBD IgG levels plateaued at 321 days (5.3 % of the peak value) and 308 days (4.8 % of the peak value) days post-vaccination, respectively (see Tables S1–S4).

The half-lives of the total antibody, anti-spike IgG, and anti-spike RBD concentrations were 94 (95 % CI: 70–143) days, 68 (95 % CI: 56–89) days, and 61 (95 % CI: 49–79) days respectively (Table 2). The mixed-effects ED model identified several independent factors associated with a faster 11 month rate of post-vaccine anti-spike total antibody decay (Table 3), including: older age, a vaccine dosing interval <35 days, and BNT162b2 (vs. mRNA-1273) vaccine type. Results examining outcomes of anti-spike and anti-RBD IgG antibody concentrations were largely consistent, except: (1) shorter vaccine dosing interval which was not significantly associated with anti-spike and anti-RBD IgG antibody decay over time; and, (2) BMI was not associated with total anti-spike antibody decay, however was associated with anti-spike and anti-RBD IgG antibody decay.

**Table 2.** Estimated half-life

Models	Random intercept (95 % CI)	Adjusted decay rates, β (95 % CI) (Days after vaccine 2)	Half-life (95 % CI)
Model 1	2.54e−14 (0.00)	−0.0032 (−0.0043, –0.0021)	94 (70, 143)
Model 2	0.17 (0.15, 0.20)	−0.0044 (−0.0054, 0.0034)	68 (56, 89)
Model 3	0.25 (0.21, 0.29)	−0.0049 (−0.0062, –0.0038)	61 (49, 79)

All models adjusted for age, vaccine type (BNT162b2 vs mRNA-1273), Sex at birth (female vs male), race (racialized vs white), tobacco use, vaccine dosing interval, BMI:18.5–25 Kg m^−2^; ‘BMI: <18.5 Kg m^−2^’; underweight, and medical history (hypertension, diabetes, asthma, and cancer)

Outcome variable for model one is Total Anti-spike Antibody Concentration;

Outcome variable for model two is Anti-Spike IgG Antibody Concentration;

Outcome variable for model three is Anti-RBD IgG Antibody Concentration.

**Table 3.** Mixed effect modelling of changes in antibody concentrations 11 months after second dose

Variables	Model 1: Total Anti-Spike antibody β (95 % CI)	Model 2: Anti-Spike IgG β (95 % CI)	Model 3: Anti-RBD IgG β (95 % CI)
**Fixed effects**			
Days after the second dose	−0.0032 (−0.0043, –0.0021)*	−0.0044 (−0.0054, 0.0034)*	−0.0049 (−0.0061, –0.0038)*
Female sex (vs. male sex)	−0.0046 (−0.11, 0.11)	−0.033 (−0.13, 0.064)	0.0024 (−0.12, 0.12)
Age (years)	−0.0060 (−0.011, –0.00058)*	−0.0079 (−0.013, –0.0031)*	−0.0070(−0.013, –0.0012)*
Racialized (vs. white)	−0.0097 (−0.18, 0.16)	0.085 (−0.057, 0.23)	0.073 (−0.099, 0.24)
(BMI: 18.5 to <25 kg m^−2^) vs. others	−0.20 (−0.49, 0.093)	0.21 (0.0054, 0.41)*	0.23 (−0.015, 0.48)
(BMI ≥25 kg m^−2^) vs. others	−0.0073 (−0.29, 0.28)	0.37 (0.15, 0.58)*	0.38 (0.13, 0.64)*
Tobacco use	−0.16 (−0.42, 0.11)	−0.12 (−0.32, 0.085)	−0.12 (−0.37, 0.12)
***Vaccine type*** (BNT162b2 vs. mRNA-1273)	−0.30 (−0.44,–0.17)*	−0.15 (−0.27, –0.031)*	−0.21 −0.36,–0.066)*
Shorter Dose 1-to-Dose 2 interval (<35 days)	−0.29 (−0.40,–0.18)*	0.022 (−0.076, 0.12)	−0.075 (−0.19, 0.044)
*Medical History*			
Hypertension	−0.18 (−0.38, 0.0088)	−0.0062 (−0.16, 0.15)	−0.038 (−0.22, 0.15)
Diabetes	−0.047 (−0.47, 0.38)	−0.078 (−0.46, 0.30)	−0.066 (−0.52, 0.39)
Asthma	0.077 (−0.061, 0.21)	0.10 (−0.019, 0.23)	0.13 (−0.021, 0.27)
Liver disease	−0.082 (−0.51, 0.34)	0.013 (−0.36, 0.39)	−0.00088 (−0.46, 0.45)
Cancer	−0.14 (−0.50, 0.22)	0.033 (−0.28, 0.35)	−0.012 (−0.40, 0.37)
**Random component**			
Intercept/constant (95 % CI)	2.54e-14 (0)	0.17 (0.15, 0.20)	0.25 (0.21, 0.29)

β (95 % CI): Effects estimate (95 % confidence interval); β values estimates were shown to two significant figures; *p* values were shown to three decimal places, se: standard error; BMI: Body Mass Index (Kg m^−2^).

*=*P*<0.05.

## Discussion

We investigated the long-term anti-spike antibody concentrations afforded by immunization of serially-tested middle-aged vaccinees with two doses of mRNA vaccine over 11 months after receiving the second vaccine who had no evidence of prior SARS-CoV-2 infection. Antibody concentrations reached maximum levels on day 21 after the second dose, subsequently declined with a half-life of 94 days, and then plateaued at a level of 1021 U ml^−1^ after approximately 10 months. Previous studies have looked at long-term antibody concentrations subsequent to other beta coronavirus infections and have found some variability with for example one study of more mild MERS-CoV infections demonstrated relatively rapid antibody seroreversion [26], while another pre-print study of antibody responses in health care workers with SARS-CoV-1 infections found persistence of detectable antibody responses beyond 12 years in most of those tested [27]. For SARS-CoV-2 infections there is also some variability in the reports but it appears that detectable antibody responses are maintained for up to 3 years [28]. Our study provides additional insights into the longer-term dynamics of SARS-CoV-2 anti-spike antibody concentration following a two dose mRNA vaccine series. Serum IgG levels following other vaccinations often will stay at a relatively stable plateau level for many years (e.g. following measles and rubella vaccination) but this duration is less clear for SARS-CoV-2 vaccination. This study demonstrates that detectable antibodies are present and have generally reached a plateau in most previously healthy individuals at 10 months post-two dose vaccination series. It remains unclear if subsequent induction of long-lived plasma cells through either re-exposure to antigen via infection or vaccination will result in a new higher steady state for anti-spike antibody levels. It is also unclear what protection, particularly against severe disease is provided during this period of plateaued antibody levels. Further longitudinal clinical studies would be needed to better understand this dynamic and may allow for antibody measurement as means for determining if booster vaccine doses are needed. We found older age, a vaccine dosing interval <35 days, and the BNT162b2 (vs. mRNA-1273) vaccine to be associated with a faster rate of post-vaccination total anti-spike antibody decay. These data may assist decision makers with the timing of booster vaccination doses. Modification of vaccination schedules may be warranted for those shown to have faster antibody decay, including older individuals, those with a shorter vaccine dosing interval, and those who received the BNT162b2 vaccine. It may also be warranted to prioritize mRNA-1273 dosing for groups that are at greater risk for rapid antibody decay.

Previous studies have investigated post-vaccine antibody decay up to 6 months after the second vaccine dose, and as well as the factors associated with antibody decline. In a study that investigated the safety and immunogenicity of two mRNA-based COVID-19 vaccines, the immune response after receiving two doses of BNT162b2 was lower in the older individuals (65–85 years) than the younger age group (18 to 55 years) [29]. Pérez-Alós *et al.* [8] modelled the waning of immunity after SARS-CoV-2 vaccination for up to 230 days after the first dose and found decay of antibody levels over time. Additionally, their study found a decrease in antibody levels among older individuals (more than 60 years) independent of previous infection. These findings are consistent with our study which demonstrates faster antibody decay among older individuals after receiving two mRNA vaccine doses. This data, in combination with previous evidence shows that older individuals are more likely to have severe COVID-19 [30, 31], and thus, supports consideration of earlier booster vaccination strategies (which have been incorporated into some clinical recommendations) [32–34].

Our results showed that patients vaccinated with BNT162b2, vs. mRNA-1273, demonstrated a faster post-vaccine antibody decay, which may have implications for booster dose timing. Previous studies have shown a similar differences between these vaccines, including mRNA-1273 demonstrating higher humoral immunogenicity [35], and a lower risk of breakthrough infections and COVID-19 related hospitalizations [36]. We also found extended mRNA vaccine dosing intervals ‘≥ 35 days’ to be associated with a slower rate of antibody decay, which is congruent with previous investigations demonstrating improved immunogenicity and vaccine effectiveness with longer, compared to standard, vaccine dosing intervals [37–40].

The optimal timing of booster vaccination remains unclear, with some advocating for annual COVID-19 vaccines [41]. Given the existing evidence demonstrating that SARS-CoV-2 antibody levels are associated with COVID-19 risk [12–14] and disease COVID-19 severity [15–17], antibody models may play a role in informing booster vaccination strategies. Our 11 month data indicates that antibody levels peak within 1 month, and then decline up to approximately 10 months. It is therefore unclear if an annual booster campaign will provide adequate protection and this will at least partially depend on whether SARS-CoV-2 will become primarily associated with seasonal infections.

## Limitations

This observation study has several limitations. There may be additional confounding variables affecting immunogenicity decay that we did not account for. Our study participants included middle-aged paramedics in Canada; results may differ in other patient populations. Antibody levels have been shown to be associated with COVID-19 clinical outcomes, however, remain surrogate markers of immunity, and thus actual clinical outcomes may differ. Also, our study did not measure and investigate other markers of immune response such as T-cell responses.

## Conclusion

Anti-spike SARS-CoV-2 antibody levels peaked within 21 days after the second mRNA vaccine, and subsequently declined, plateauing at approximately 10 months after the second dose. Older age, shorter vaccine dosing interval (< 35 days), and the BNT162b2 vaccine were associated with a faster rate of post-vaccination antibody decay. These findings may inform booster frequency, including patient-specific schedules.

## Supplementary Data

Supplementary material 1
