# Supplementary material for: Eleven-month SARS-CoV-2 binding antibody decay, and associated factors, among mRNA vaccinees: implications for booster vaccination
Source: Access Microbiol. 2023 Nov 28;5(11):000678.v3. doi: 10.1099/acmi.0.000678.v3 (PMC10702380; doi:10.1099/acmi.0.000678.v3)
Supplement: Supplementary material 1 [file acmi-5-678.v3-s001.pdf]

SUPPLEMENTARY MATERIALS

Supplementary Methods

The Double exponential decay (DED) model is expressed mathematically as:

$$\boldsymbol{Ab_{levels}} = \boldsymbol{Plateau} + \boldsymbol{SpanFast} * \mathbf{exp}(-\mathbf{KFast} * \mathbf{T}) + \mathbf{SpanSlow} * \mathbf{exp}(-\mathbf{KSlow} * \mathbf{T})$$

Where, $\boldsymbol{SpanFast} = (\boldsymbol{Y_0} - \boldsymbol{plateau}) * \boldsymbol{PercentFast} * \mathbf{0.01}$

$$\boldsymbol{SpanSlow} = (\boldsymbol{Y_0} - \boldsymbol{Plateau}) * (\mathbf{100} - \boldsymbol{PercentFast}) * \mathbf{0.01}$$

$\boldsymbol{Y_0}$ is antibody levels ($\mathbf{Ab}_{levels}$) when the time "**T**" is zero. The ***Plateau*** is the antibody level at the infinite times; ***KFast*** and ***KSlow*** are the two rate constants expressed as the inverse of the time "**T**" in the x-axis; ***TauFast*** and ***TauSlow*** are the two-time constants and they are estimated as the inverse of the rate constants (i.e. **1/KFast** and **1/Kslow**);

Half-life (fast) and ***Half-life (slow)*** are the time units of the Time "**T**" and are computed as (**ln2/K**); and ***PercentFast*** is the fraction of the **span** (defined as the distance between $\boldsymbol{Y_0}$ and ***Plateau*** point)[24].

Supplementary Figures

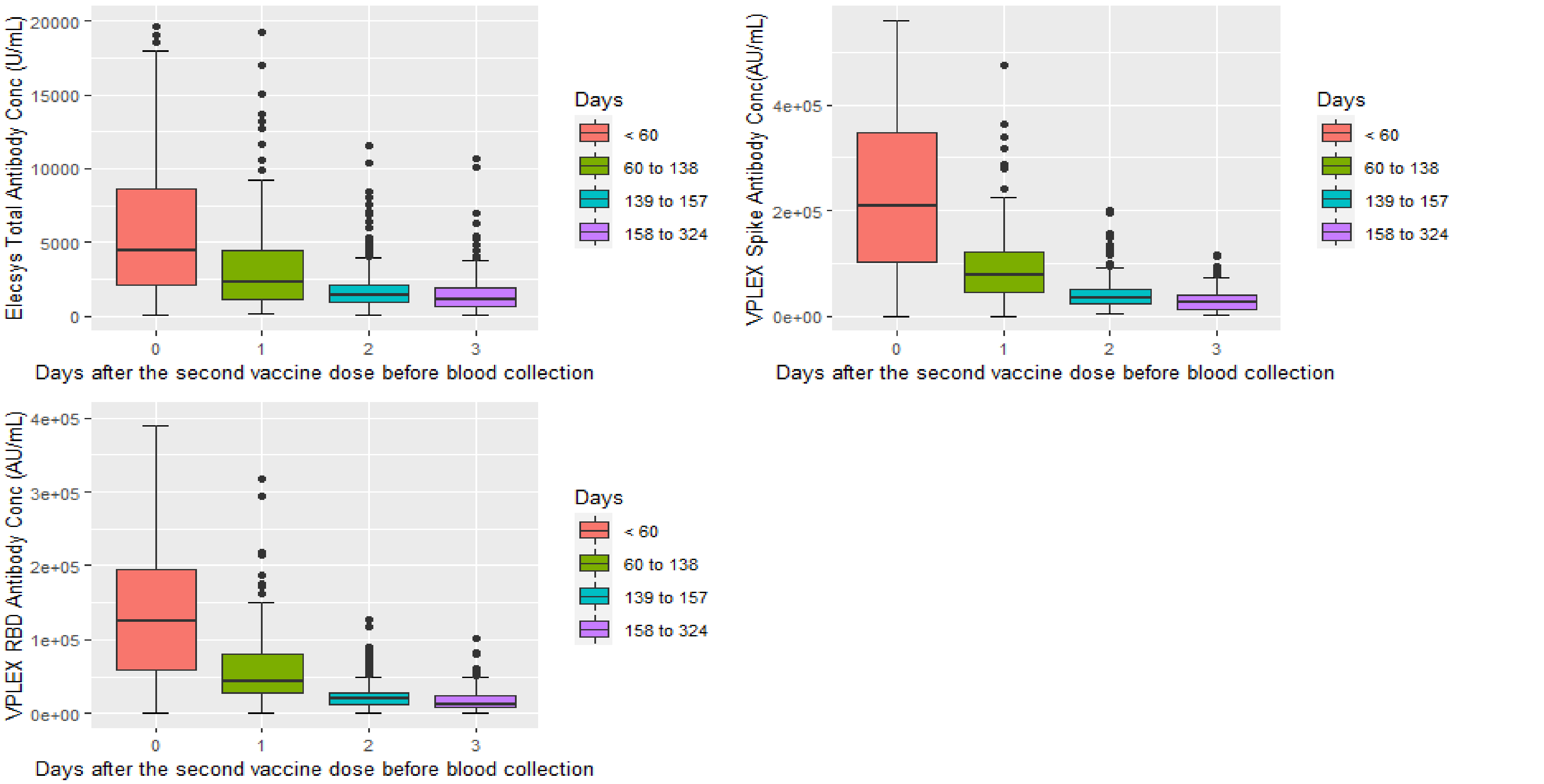

Figure S1: Box and whisker plot of antibody concentration decay by days after second vaccine (categorized into four quartiles). Days (time) was calculated as the time interval between the date of the second dose and the date of first and second blood collections.

Supplementary Tables

Table S1: Estimated peak antibody concentrations.

**Antibody concentration**	**Peak antibody value**	**Day (P)**	**Plateau value**	**Day (L)**
Total Anti-Spike Antibody concentration	9,042	21	1021	288
Anti-Spike IgG Concentration	323,980	21	17287	321
Anti-RBD IgG Concentration	249,051	21	11909	308

Day (P): the day the peak antibody levels were recorded; *Day (L)*: the day/time the lowest antibody level was recorded. Lowest IgG value defined as the value equal to when the antibody levels declined to less than 5% of the peak antibody value.

Table S2: Table showing results from the DED for Total antibody concentration (U/mL)

Two phase decay	
Best-fit values	
Y0	9741
**Plateau**	**1021**
PercentFast	77.73
KFast	0.01835
KSlow	0.007189
Half Life (Slow)	96.42
Half Life (Fast)	37.78
Tau (slow)	139.1
Tau (fast)	54.51
Rate constant ratio	2.552
Goodness of Fit	
Robust Sum of Squares	32.63
RSDR	320.0
Constraints	
PercentFast	0 < PercentFast < 100
KFast	KFast > 1*KSlow
KSlow	KSlow > 0

Table S3: Table showing results from the DED model for anti-spike IgG (AU/mL)

Two phase decay	Hit constraint
Best-fit values	
Y0	311950
**Plateau**	**17287**
PercentFast	~ 25.43
KFast	~ 0.01488
KSlow	0.01488
Half Life (Slow)	46.58
Half Life (Fast)	~ 46.58
Tau (slow)	67.21
Tau (fast)	~ 67.21
Rate constant ratio	~ 1.000
Goodness of Fit	
Robust Sum of Squares	40.60
RSDR	6108
Constraints	
PercentFast	0 < PercentFast < 100
KFast	KFast > 1*KSlow
KSlow	KSlow > 0

Table S4: Table showing results from the DED model for anti-RBD IgG (AU/mL)

Two phase decay	
Best-fit values	
Y0	244272
**Plateau**	**11909**
PercentFast	86.06
KFast	0.01716
KSlow	0.01716
Half Life (Slow)	40.40
Half Life (Fast)	40.40
Tau (slow)	58.29
Tau (fast)	58.29
Rate constant ratio	1.000
Goodness of Fit	
Robust Sum of Squares	37.81
RSDR	5875
Constraints	
PercentFast	0 < PercentFast < 100
KFast	KFast > 1*KSlow
KSlow	KSlow > 0
